# Selective recovery of platinum from spent autocatalyst solution by thiourea modified magnetic biocarbons

**DOI:** 10.1038/s41598-021-98118-1

**Published:** 2021-09-29

**Authors:** Shao-Yi Lo, Wahid Dianbudiyanto, Shou-Heng Liu

**Affiliations:** 1 Department of Environmental Engineering, National Cheng Kung University, Tainan, 70101 Taiwan; 2 Department of Biology, Faculty of Science and Technology, Universitas Airlangga, Surabaya, 60115 Indonesia

**Keywords:** Environmental sciences, Engineering, Materials science

## Abstract

The precious platinum group metals distributed in urban industrial products should be recycled because of their rapid decline in the contents through excessive mining. In this work, thiourea modified magnetic biocarbons are prepared via an energy-efficient microwave-assisted activation and assessed as potential adsorbents to recover platinum ions (i.e., Pt(IV)) from dilute waste solution. The physicochemical properties of prepared biocarbons are characterized by a series of spectroscopic and analytic instruments. The adsorption performance of biocarbons is carried out by using batch tests. Consequently, the maximum adsorption capacity of Pt(IV) observed for adsorbents is ca. 42.8 mg g^−1^ at pH = 2 and 328 K. Both adsorption kinetics and isotherm data of Pt(IV) on the adsorbents are fitted better with non-linear pseudo second-order model and Freundlich isotherm, respectively. Moreover, the thermodynamic parameters suggest that the Pt(IV) adsorption is endothermic and spontaneous. Most importantly, the adsorbents exhibit high selectivity toward Pt(IV) adsorption and preserve ca. 96.9% of adsorption capacity after six cyclic runs. After adsorption, the regeneration of the prepared adsorbents can be effectively attained by using 1 M thiourea/2% HCl mixed solution as an eluent. Combined the data from Fourier transform infrared and X-ray photoelectron spectroscopies, the mechanisms for Pt(IV) adsorption are governed by Pt–S bond between Pt(IV) and thiourea as well as the electrostatic attraction between anionic PtCl_6_^2−^ and cationic functional groups of adsorbents. The superior Pt(IV) recovery and sustainable features allow the thiourea modified magnetic biocarbon as a potential adsorbent to recycle noble metals from spent autocatalyst solution.

## Introduction

Due to the industrial revolution, commercial products have been manufactured by the intense usage of metals. Among them, platinum group metals (PGMs) have been widely used in energy devices, catalysts, automobile and petrochemical industrials in recent years because of their specific physicochemical properties, e.g., high melting point, electrothermal stability, excellent corrosion resistance, and superior catalytic activity^1–3^. The PGMs are hard to be replaced by other metals due to their aforementioned unique properties. Therefore, industrial wastes including spent catalysts and electronic wastes have been regarded as one of the secondary PGMs resources^4–6^. From the viewpoints of urban mining, it is essential to develop effective methods to separate and recycle PGMs from above-mentioned waste streams.

The recovery technologies of PGMs can be mainly divided into pyrometallurgy and hydrometallurgy. Pyrometallurgy is a metallurgical process that extracts metals from raw materials at high temperatures by the sequence of ore preparation, smelting and refining^7–9^. Hydrometallurgy includes solvent extraction, precipitation and ion exchange that extract metals by chemicals^10–12^. However, these methods may suffer from the high cost, energy consumption and secondary pollution^13,14^. For example, the recovery of PGMs was reported by using organic solvents (e.g., toxic cyanide^15^/aqua regia^16^/chloroform^17^, ion exchange membrane^18,19^ and ionic liquids^20–22^. It was mentioned that the adsorption technique was one of economical and eco-friendly strategies for the recycle of PGMs^23–27^. Among various adsorbents, the biocarbons exhibit potential benefits in the environmental remediation, energy conversion and storage^28–32^ due to their abundant functional groups, high mechanical and chemical stability and renewable sustainability. For instance, the activated carbons and carbon nanotubes were prepared from low-value biomasses (i.e., alkali silicate herbaceous and brewer's spent grain biomasses) via two‐stage activation for wastewater remediation^33,34^. The results demonstrate the circular economy concept by upcycling biowaste feedstocks for value-added applications. Also, the simple chemical modification of biocarbons can further enhance the adsorption capacity and selectivity toward PGMs from a complex solution. The efficient applications of sulfur-containing functional groups toward PGMs recovery were reported^35–40^. However, very limited studies on the thiourea/biocarbons as adsorbents for the adsorption of PGMs can be found.

It is estimated^41^ that the demand for coffee around the world has been increasing year by year. Therefore, the spent coffee grounds (SCGs) are intensively produced and mostly used as fertilizers in the agriculture or fume adsorbents in the household. In terms of zero-waste hierarchy for management of biomass (i.e., SCGs), thermochemical conversion of SCGs may be a better route compared to other methods^42^. To further enable the volarization of SCGs, in this study, the thiourea-modified magnetic biocarbons (denoted as Tu–N–SCG–C–A), which can be easily recovered with an external magnetic field, were prepared from SCGs by using physical (i.e., CO_2_) and chemical (i.e., KOH) activation under microwave irradiation. The surface of biocarbons was further nitrogen-doped by microwave NH_3_ ammoxidation^43–45^. Then, these biocarbons were cross-linked with thiourea functional groups via glutaraldehyde. The physicochemical properties of Tu–N–SCG–C–A were examined by various analytic methods [i.e., N_2_ adsorption–desorption isotherms and elemental analysis (EA)] and spectroscopies [i.e., X-ray diffraction (XRD), X-ray photoelectron spectroscopy (XPS), transmission electron microscopy (TEM) and Fourier transform infrared spectroscopy (FTIR)]. The objectives of this research were to (1) synthesize and characterize the prepared Tu–N–SCG–C–A with various techniques and evaluate its feasibility to act as an adsorbent in the recovery of Pt(IV) ions, (2) explore the adsorption kinetic, isotherm, thermodynamic studies of Pt(IV) ions and study the effects of adsorbent dosages, pH and competitive adsorption, (3) and identify the plausible adsorption mechanisms of Pt(IV) onto Tu–N–SCG–C–A.

## Materials and methods

### Chemicals

The SCGs which contained hemicellulose (30–40 wt%), lignin (25–33 wt%), cellulose (8.6–13.3 wt%) and protein (6.7–13.6 wt%)^46^ were collected from cafeterias in Tainan city (Taiwan). To eliminate the impurities, the SCGs were washed with boiled and distilled water till the colour of the water was no longer turbid, followed by drying in an oven at 60 °C for 24 h. Afterward, the pre-treated SCGs would be stored in a moisture-proof box for further biocarbon preparation. In this work, all chemicals were analytical reagent grade, including (1) potassium hydroxide (KOH) and ammonia solution (NH_3_) from Sigma-Aldrich; (2) iron(III) chloride (FeCl_3_) and thiourea from Acros; (3) iron(II) sulfate heptahydrate (FeSO_4_·7H_2_O) and glutaraldehyde from Showa; and (4) platinum and palladium ICP standard were obtained from Merck.

### Preparation of magnetic biocarbons

Firstly, the SCGs were mixed with 0.2 M of FeCl_3_ and FeSO_4_·7H_2_O solution (2:1) in a ratio of 1:15. The mixture was well mixed at 60 °C for 2 h, followed by adding 2.5 M of NH_3_ solution slowly. The pH was adjusted to 12 and keep stirring for 1 h at 70 °C to generate Fe_3_O_4_ on SCGs. After drying at 60 °C, the samples were then carbonized at 700 °C in the atmosphere of CO_2_ (flowrate = 500 mL min^−1^). The resultant samples were denoted as SCG–C. To further activate the biocarbons, the microwave-assisted KOH activation was performed. About 1 g of prepared SCG–C samples were mixed with 3 g of KOH in 2.5 mL of deionized (DI) water solution. After drying, the samples were heated under 700 W microwave oven (Milestone, Ethos Easy) for 10 min in a N_2_ flow of 100 mL min^−1^. After that, the activated samples were washed with DI water until the filtrate reaching pH = 7, which was named as SCG–C–A. In order to have more active sites in the samples, N-doping treatment was carried out by irradiating 700 W microwave under an NH_3_ atmosphere for 10 min. The obtained samples were denoted as N–SCG–C–A. Thiourea-modified magnetic biocarbons were further prepared. Firstly, ca. 2.5 g of thiourea was dissolved in 50 mL water and glutaraldehyde was used as the crosslinker at 50 °C for 3 h in a reflux device. Afterward, ca. 1 g of the N–SCG–C–A sample was added into the above mixture at 70 °C and kept stirring for 8 h. The obtained sample was denoted as Tu–N–SCG–C–A.

### Characterizations of magnetic biocarbons

The XRD patterns were recorded by using a D8 DISCOVER (Bruker AXS Gmbh, Germany) diffractometer (Cu Kα, *λ* = 1.541 Å) operated at 40 kV and 40 mA. The identification of functional groups on biocarbons was performed by using a FTIR spectroscopy (Bruker TENSOR 27). The elemental compositions of biocarbons were revealed by elemental analyzer (Elementar vario EL III). The porosity and the Brunauer–Emmett–Teller (BET) surface area were measured by using a volumetric analyser (Micromeritics ASAP 2020). The surface elemental speciations of biocarbons were analysed by employing XPS (PHI 5000 Versa Probe). The microstructures and morphologies of biocarbons were characterized by using TEM (JEOL-2100F).

### Batch adsorption experiments

Firstly, 50 mg L^−1^ stock solution of Pt(IV) was prepared by respectively dissolving certain amounts of H_2_PtCl_6_·5H_2_O in 0.1 M HCl solution. A series of adsorption tests including the effect of adsorbent dosages, pH effect (pH = 2–8), kinetics, adsorption isotherms, thermodynamics, adsorption selectivity, adsorption–desorption and reusability were evaluated. Typically, 0.25 g of adsorbents was introduced into a flask with 100 mL of Pt(IV) solution at 25 °C, followed by placing in a temperature-controllable shaker at 175 rpm for adsoprtion equilibrium. After adsorption, the concentrations of precious metals in the filtrate were analyzed by an inductively coupled plasma-optical emission spectrometer (ICP-OES, JY ULTIMA 2000). The equilibrium adsorption capacities (q_e_, mg g^−1^) of Pt(IV) on biocarbons were estimated by using the mathematical Eq. (1):

1 $$ {\text{q}}_{{\text{e}}} = \frac{{\left( {{\text{C}}_{0} - {\text{C}}_{{\text{e}}} } \right) \times {\text{V}}}}{{\text{M}}} $$

where C_0_ and C_e_ represent the initial and equilibrium metal concentrations (mg L^−1^), V denotes the solution volume (L) and M signifies the initial mass (g) of adsorbents.

The spent biocarbons were regenerated by using different mixtures of ethylenediaminetetraacetic acid (EDTA)/HCl and thiourea/HCl solution to desorb metal ions. The selectivity adsorption of Pt (IV) onto prepared biocarbons was investigated in the presence of the simulated metal ions (e.g., Pt(IV), Ni(II), Cu(II), Zn(II) and Pb(II)) of spent catalysts from catalytic converter). The reusability of the prepared biocarbons for adsorption and desorption of metal ions was carried out. The regenerated adsorbents were washed with DI water for several times and then dried at 70 °C overnight before they were used for next batch adsorption.

## Results and discussion

### Physicochemical properties of prepared biocarbons

**Figure 1:**
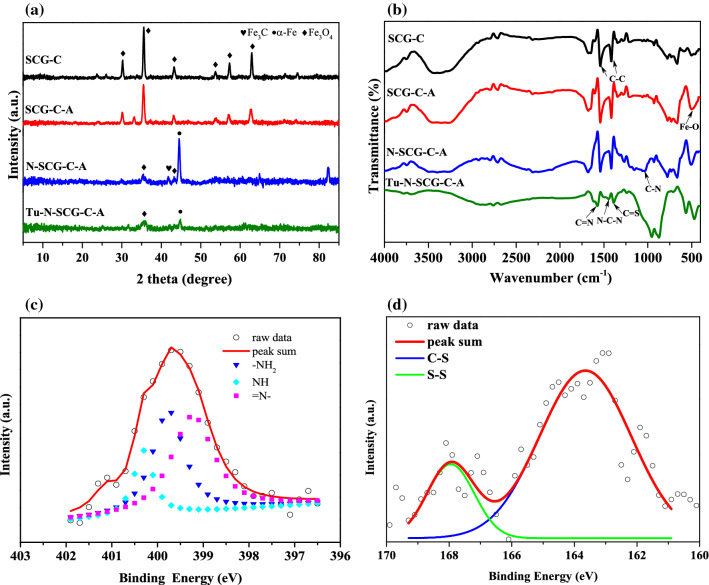
(**a**) X-ray diffraction patterns, (**b**) FTIR spectra of various samples, (**c**) XPS N 1 s and (**d**) S 2*p* spectra of Tu–N–SCG–C–A.

**Figure 2:**
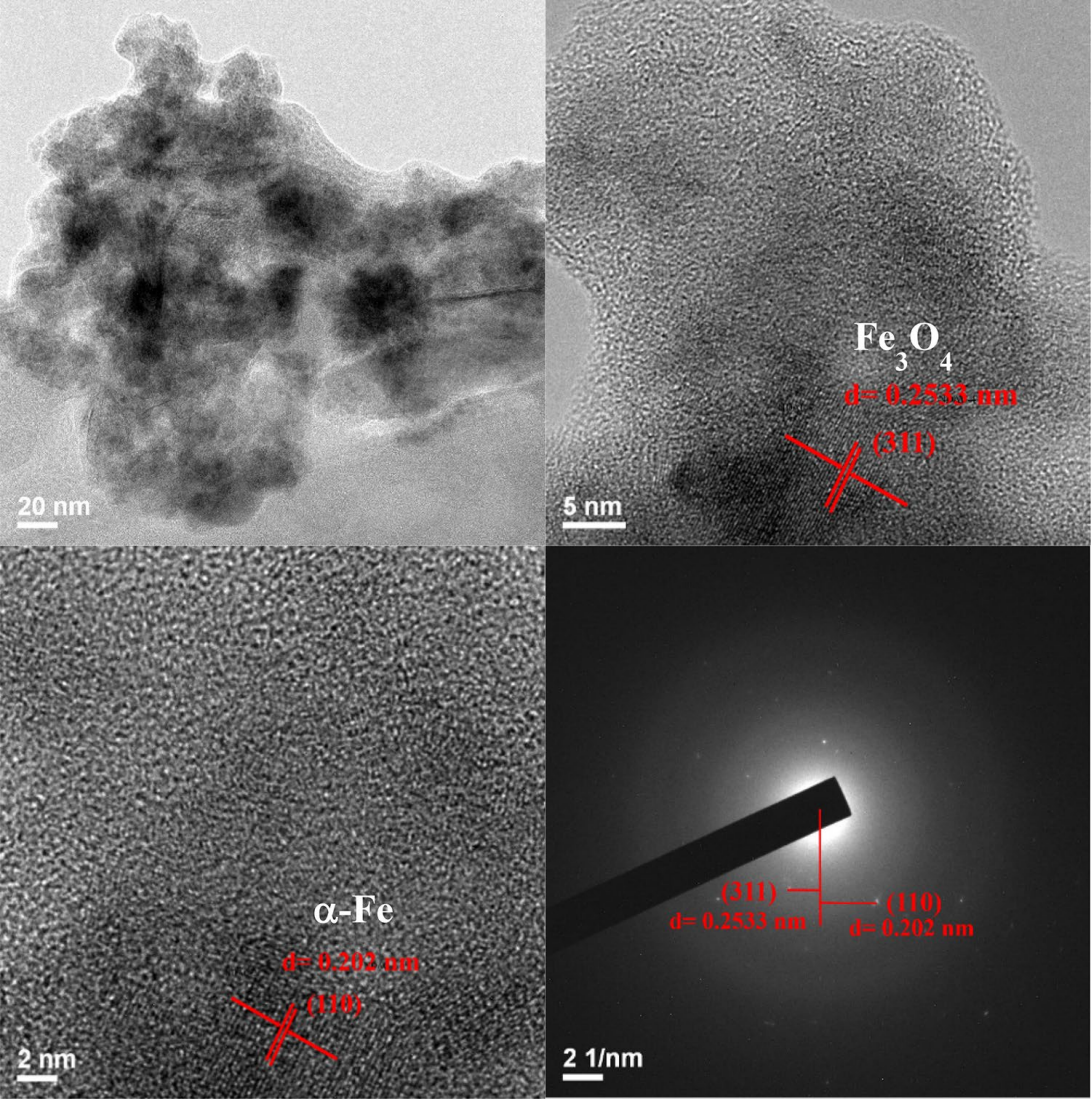
High-resolution TEM and SAED images of Tu–N–SCG–C–A.

As shown in Fig. 1a, the XRD peaks at 2θ of 30.3°, 35.7°, 43.4°, 53.8°, 57.0° and 62.6° observed for SCG-C are the characteristics of (220), (311), (400), (422), (511) and (440) basal planes of magnetite (Fe_3_O_4_) (JCPDS No. 19-0629)^47,48^. Upon activating SCG-C with microwave-assisted KOH process (i.e., SCG–C–A), the structure of Fe_3_O_4_ is almost intact. However, the peak intensity of Fe_3_O_4_ observed for N–SCG–C–A is noticeably decreased after microwave-assisted N-doping process. At the same time, the peaks at 2θ = 44.8° and 41.8° are ascribed to the formation of α-Fe and Fe_3_C, respectively. Moreover, the crystalline phases of Fe_3_O_4_ and α-Fe still can be found for Tu–N–SCG–C–A. The magnetic crystalline of iron in the Tu-N-SCG-C-A biocarbons can be further verified via TEM images as can be seen in Fig. 2. The well-identified Fe_3_O_4_ (311) and α-Fe (110) lattice fringes with d-spacings of 0.253 and 0.202 nm^49,50^, respectively can be observed. Furthermore, selected area electron diffraction (SAED) pattern confirms the above results, which are in good accordance with aforementioned XRD.

The FTIR spectra were used to understand the functional groups on the biocarbons. As displayed in Fig. 1b, the FTIR spectra of SCG–C and SCG–C–A show the similar patterns, which have broad bands at ca. 3343 cm^−1^ attributing to the stretching vibration of hydroxyl functional groups (i.e., –OH)^51^ and two peaks at ca. 1600 and 1400 cm^−1^ respectively relating to the vibrations of aromatic rings and the C–C stretching of aromatic groups^52^. Also, the shoulder at 564 cm^−1^ is attributed to the Fe–O–Fe vibration of magnetite phase, again indicating the existence of Fe_3_O_4_ on the biocarbons^53^. The N–SCG–C–A biocarbons have broad features in the range of 3200–3700 cm^−1^, indicating the presence of –OH and –NH groups. In addition, the peak at ca. 1038 cm^−1^ is attributed to vibration of C–N groups, implying the successful N-doping on the biocarbons^54^. The peaks observed for Tu–N–SCG–C–A biocarbons at ca. 1650, 1530 and 1378 cm^−1^ are assigned to the C=N, N–C–N and C=S stretching vibrations, respectively. The above FTIR features suggest that the thiourea has been successfully modified on the magnetic biocarbons^55–57^. The amounts of N and S elements on the prepared biocarbons are summarized in Table 1. The N contents are decreased upon the carbonization and activation process. After microwave-assisted N-doping, the N amounts are increased. It is worthy to note that the S content in the Tu–N–SCG–C–A is increased to 0.49 wt% which is at least three times that of N–SCG–C–A, again proofing the effective modification thiourea onto N–SCG–C–A biocarbons.

**Table 1:** Textural properties of spent coffee grounds and their derived biocarbons.

Sample	S_BET_ (m^2^/g)	V_t_ (cm^3^/g)	N (%)	S (%)
SCG	–	–	2.03	0.06
SCG–C	256.6	0.18	1.76	0.26
SCG–C–A	320.9	0.18	0.29	0.09
N–SCG–C–A	202.6	0.15	0.75	0.13
Tu–N–SCG–C–A	13.8	0.05	1.79	0.49

The porous structures of prepared biocarbons were studied by N_2_ adsorption/desorption isotherms. In Table 1, the BET surface areas and pore volumes of SCG–C and SCG–C–A are remarkably increased as compared to that of SCG. However, the surface area of N–SCG–C–A is reduced, indicating that the N-doping via microwave-assisted treatments may alter the structure. Because the pores of N–SCG–C–A could be occupied via the glutaraldehyde cross-linking reaction between thiourea and SCGs, the surface area (13.8 m^2^ g^−1^) and pore volume (0.05 cm^3^ g^−1^) of Tu–N–SCG–C–A are decreased compared to those of N–SCG–C–A.

The high-resolution XPS N 1 s fitted spectrum of Tu–N–SCG–C–A is shown in Fig. 1c. The peaks at 399.0, 399.7 and 400.3 eV are assigned to nitrogen in the binding structure of =N–, –NH_2_ and N–H, respectively which indicates that the possible formation of imine^57^ due to the cross-linking reaction via glutaraldehyde between SCG and thiourea (also discuss later). The high-resolution XPS S 2*p* spectrum of Tu–N–SCG–C–A is shown in Fig. 1d. The S 2*p* peak is composed of S 2*p*_3/2_ and S 2*p*_1/2_ at 163.6 and 167.9 eV, respectively, suggesting the probable existence of C–S species^40^.

## Adsorption performance of Tu–N–SCG–C–A

### Effect of dosages and pH

**Figure 3:**
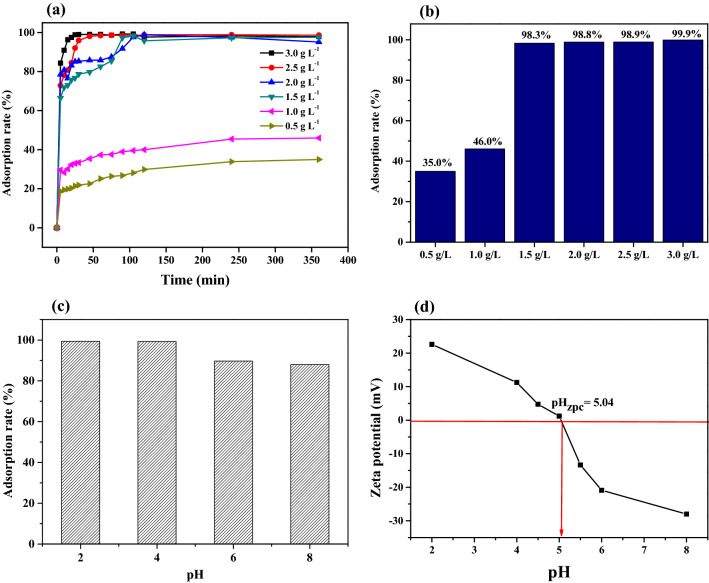
(**a**), (**b**) Effect of dosage on Pt(IV) adsorption capacities, (**c**) effect of pH on Pt(IV) adsorption and (**d**) zeta potential of Tu–N–SCG–C–A.

The effect of adsorbent dosages (0.5–3.0 g L^−1^) on the adsorption of Pt(IV) (50 mg L^−1^) was evaluated by batch adsorption tests. Figure 3a, b illustrate that the Pt(IV) ions can be adsorbed onto the biocarbons rapidly in the initial stage and reach the equilbrium with time. Upon loading the dosage to 1.5 g L^−1^, the adsorption rate can be increased to 98.3%. However, it takes ca. 90 min to reach the equilbrium for the dosage of 1.5 g L^−1^. For the dosage of 2.5 g L^−1^, the equilibrium can be achieved within 45 min. The increased dosages can provide more availability of surface active sites on the adsorbents^58^. The above results show that the dosage of 2.5 g L^−1^ is effective in terms of the maximum adsorption efficiency and rapid rate to equilibrium.

The influence of pH on the adsorption efficiency of Tu–N–SCG–C–A biocarbons between pH 2.0–8.0 is presented in Fig. 3c. It can be found that the higher values of pH, the lower of adsorption capacity of Pt(IV) ions. The highest removal efficiency of 99.4% Pt(IV) can be observed for Tu–N–SCG–C–A at pH = 2.0. However, the adsorption of Pt(IV) decreases to 88.0% at pH value = 8.0. The effect of pH on the adsorption of Pt(IV) could be explained by the point zero charge (pH_PZC_), as shown in Fig. 3d. The pH_PZC_ of Tu-N-SCG-C-A biocarbons is ca. 5.04. Whenever pH value is less than 5.04, the surface of adsorbents is charged positively, while charged negatively at pH > 5.04. It was reported that the speciation of Pt(IV) varied with the concentration of Cl^-^ and pH^59^. In the range of low pH, the dominant species of Pt(IV) are [PtCl_6_]^2−^ and [PtCl_5_(H_2_O)]^−^^60^. The zeta potentials of Tu–N–SCG–C–A biocarbons become more negative as the pH > 5.04 probably due to the deposition of more OH^−^ on the Tu–N–SCG–C–A surface, which the electrostatic repulsion may occur. On the contrary, more NH_3_^+^ are formed on the Tu–N–SCG–C–A biocarbons. Thus, the surface charge of Tu–N–SCG–C–A is positive upon the pH < 5.04. Consequently, the protanation of amine groups on the Tu–N–SCG–C–A biocarbons results in an electrostatic attraction of anionic metal complexes^36,61^.

### Kinetics of Pt adsorption

**Figure 4:**
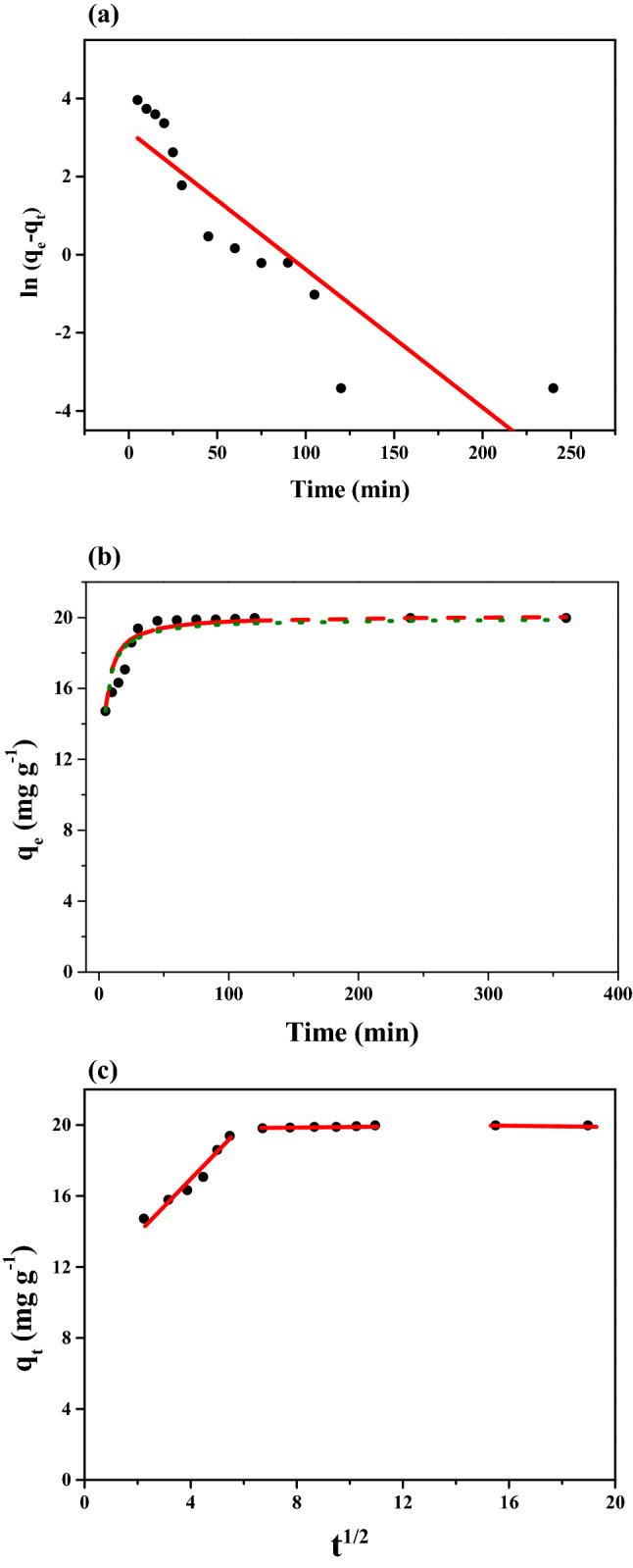
(**a**) Pseudo first order, (**b**) comparison of the estimation of linear and non-linear regressions of pseudo-second order model for Tu–N–SCG–C–A (red dash line: non-linear regressions; green dot line: linear regressions) (**c**) adsorption kinetics of intraparticle diffusion model (operating conditions: 250 mg of biocarbons and 50 mL of 50 mg L^−1^ Pt(IV) solution, pH = 2).

In order to study the kinetics and the possible mechanisms of adsorption process, the experimental data were elucidated by fitting with three kinetic models, namely, pseudo-first-order, pseudo-second-order and intraparticle diffusion models. Tables 2 and 3 list the fitting parameters of pseudo-first-order and pseudo-second-order kinetic models. The correlation coefficient (R^2^) and normalized standard deviation (SD: $\frac{{\left( \% \right) = 100x\sqrt {\sum \left[ {\frac{{(q_{t, exp} - q_{t,cal)} }}{{q_{t, exp} }}} \right]^{2} } }}{{\left( {n - 1} \right)}}$) are used to evaluate the fittness of these models. Compared to the pseudo-first-order model, the higher values of R^2^, the lower values of SD and better fitted between experimental and calculated q_e_ values obtained from both linear and non-linear pseudo-second-order kinetic models indicate that the adsorption process of Pt(IV) onto Tu–N–SCG–C–A is reasonably interpreted on the basis of pseudo-second-order kinetic model. The above result is in good accordance with plots in Fig. 4a, b. The pseudo-second kinetic order model is based on the assumption that the rate-limiting step may be a chemisorption involving valance forces through sharing or exchanging electrons between adsorbent and adsorbate^62^. The theoretical adsorption capacity of adsorbents is calculated to be 20.12 mg g^−1^ for Pt(IV). The fitting results have better correlation to linear model with R^2^ = 0.9999. However, the values of SD of non-linear model are lower compared to those of linear model, indicating that the non-linear model is the better expression to predict the kinetics of Pt(IV) sorption onto Tu–N–SCG–C–A. The fitting results of both linear and non-linear pseudo-second-order kinetic model are poltted in Fig. 4b. It also can be confirmed that the Pt(IV) adsorption onto Tu–N–SCG–C–A have a better prediction by using the non-linear pseudo-second kinetic order expression. Based on Weber and Morris’s theory^63^, intraparticle diffusion also can be used to investigate the adsorption kinectics. It can be seen from Fig. 4c that the plots of q_t_ versus t^1/2^ consist of more than one step of adsorption process with three distinct reigons. The adsorption process of Pt(IV) on adsorbents could be described as three consecutive steps: (I) the bulk and film diffusion of Pt(IV) in solution; (II) internal diffusion on the surface of adsorbent; and (III) chemical interaction at the surface of adsorbents^64,65^. The slopes of three adsorption steps are decreased upon the increase of adsorption time, indicating that the adsorption rates are decreased due to the decrease of adsorption sites. At the same time, it is observed that the intercepts of three steps are increased by following the order: region III > region II > region I. The aforementioned results suggest that the chemical interaction at the surface of adsorbents is the rate-limiting step.

**Table 2:** Parameters of Pseudo-first-order kinetic model for Pt(IV) adsorption on Tu–N–SCG–C–A.

q_e,exp_ (mg g^−1^)	Pseudo first order			
	${\text{k}}_{1}$(min^−1^)	q_e_,_cal._ (mg g^−1^)	R^2^	SD(%)
19.97	0.035	23.65	0.8011	2.56

**Table 3:** Parameters of Pseudo-second-order-kinetic model for Pt(IV) adsorption on Tu–N–SCG–C–A.

q_e,exp_ (mg g^−1^)	Linear			
	${\text{k}}_{2}$(g mg^−1^ min^−1^)	q_e_, _cal._ (mg g^−1^)	R^2^	SD (%)
19.97	2.81 × 10^−2^	20.12	0.9999	1.34

q_e,exp_ (mg g^−1^)	Non-linear			
	${\text{k}}_{2}$(g mg^−1^ min^−1^)	q_e_, _cal._ (mg g^−1^)	R^2^	SD (%)
19.97	5 × 10^–2^	20.12	0.9999	1.27

### Adsorption isotherms and thermodynamics

**Figure 5:**
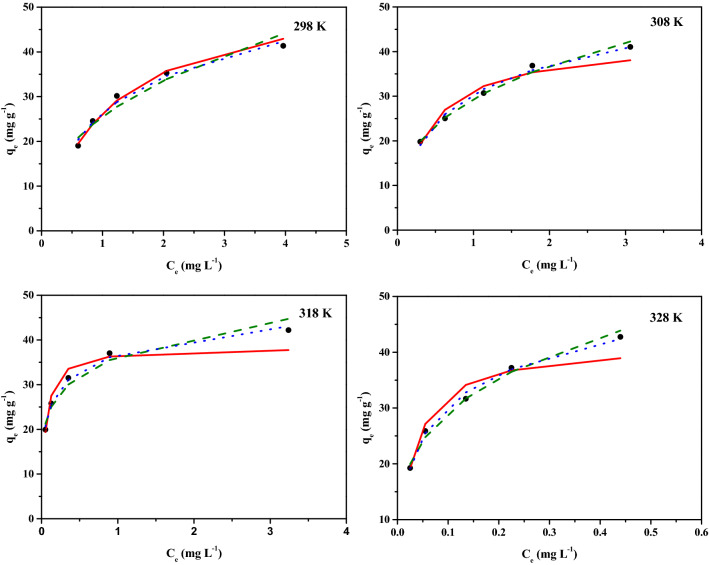
Adsorption isotherms of Pt(IV) on Tu–N–SCG–C–A at different temperatures (298–328 K). Operating conditions: 250 mg of biocarbons and 50 mL of 50 mg L^−1^ Pt(IV) solution, pH = 2) (red solid line: Langmuir model; green dash line: Freundlich model; blue dot line: Temkin model).

In order to investigate the reaction behavior of adsorbents and adsorbates, the adsorption isotherms were employed. In this study, three adsorption isotherms at 298, 308, 318 and 328 K including Langmuir, Freundlich, and Temkin adsorption isotherms models were used to evaluate the adsorption of Tu-N-SCG-C-A. As shown in Fig. 5, the experimental values of q_e_ increase gradually as the initial concentrations of Pt(IV) increase. Furthermore, the equilibrium concentrations gradually increase as the temperatures increase. The fitting results of adsorption isotherm models and their derived parameters are listed in Table 4. With the similar R^2^ observed for Langmuir and Freundlich models, the SD values for Pt(IV) adsorption of Freundlich model are smaller than those of Langmuir model. As reported previously^52^, the evaluation of error analysis by using SD values should be better than R^2^ values for adopting the best-fitted isotherm model. Hence, the obtained results are indicative that both of the adsorption of Pt(IV) onto Tu–N–SCG–C–A should follow Freundlich adsorption model, suggesting that the multilayer adsorption may be occurred on the Tu–N–SCG–C–A. Meanwhile, the values of n which can be expressed as adsorption intensity between adsorbents and the adsorbates are found to be greater than one, indicating the favorable adsorption of Pt(IV) onto Tu–N–SCG–C–A^66^. Table S1 shows the Pt(IV) adsorption capacities of Tu–N–SCG–C–A and previously reported adsorbents. The adsorption performance is mostly associated with the adsorbent preparation method and experimental conditions (e.g., adsorbent dosages, temperature, time, pH and agitation). The performance of fabricated Tu–N–SCG–C–A is analogous to those of earlier reported adsorbents. It is noteworthy that the microwave-assisted activation in the adsorbent preparation may provide time and energy-efficient route for possible applications.

**Table 4:** Parameters of Adsorption Isotherms including Langmuir, Freundlich and Temkin Models for Pt(IV) on Tu–N–SCG–C–A.

Temp	Langmuir					Freundlich				Temkin		
	q_m, exp_ (mg g^−1^)	q_m_ (mg g^−1^)	${\text{K}}_{{\text{L}}}$(L mg^−1^)	R^2^	SD (%)	${\text{K}}_{F}$(L mg^−1^)	n	R^2^	SD (%)	${\text{K}}_{T}$(L mg^−1^)	R^2^	SD (%)
298 K	41.35	54.56	0.92	0.9896	1.62	25.51	2.52	0.9412	1.14	11.65	0.9830	2.24
308 K	41.05	42.63	2.75	0.9610	3.18	29.40	3.08	0.9927	0.35	9.47	0.9888	1.69
318 K	42.20	38.35	20.20	0.9550	3.64	36.23	5.57	0.9649	0.83	5.41	0.9924	1.26
328 K	42.76	41.51	35.52	0.9740	3.24	54.99	3.64	0.9892	0.50	8.12	0.9951	1.03

**Figure 6:**
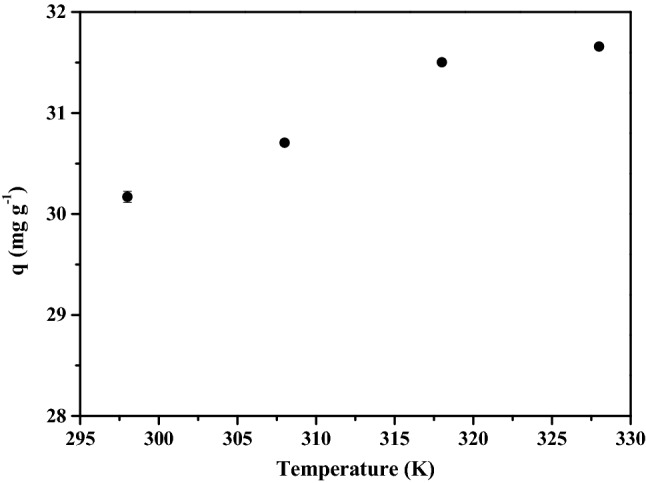
Effect of temperatures (298–328 K) on the adsorption capacities of Pt(IV) on Tu–N–SCG–C–A. Operating conditions: 250 mg of biocarbons and 50 mL of 80 mg L^−1^ Pt(IV) solution, pH = 2.

The thermodynamic parameters (ΔH°, ΔS° and ΔG°) observed for adsorption of Pt(IV) onto Tu–N–SCG–C–A at four different temperatures (298, 308, 318 and 328 K) were calculated and summarized in Table 5. The values of ΔG° are negative, showing that the adsorption processes of Tu–N–SCG–C–A are spontaneous. In particular, ΔG° becomes more negative when the temperatures increase from 298 to 328 K, indicating that the adsorption process is more favorable at higher temperatures. As confrimed in Fig. 6, the adsorption capacities of Pt(IV) are increased as the temperatures are elevated from 298 to 328 K. As a result, the values of ΔH° are calculated to be 20.27 kJ mol^−1^ for Pt(IV), revealing that the interaction between Pt(IV) and the Tu–N–SCG–C–A is an endothermal adsorption process. As such, a large amount of heat is consumed to transfer metal ions from aqueous to the solid phase. Also, it is in line with the increase of Pt(IV) adsorption capacity when the temperature is increased. Moreover, the positive value of ΔS° (94.39 J mol^−1^ K^−1^) is indicative of favorable Pt(IV) adsorption due to the increased randomness at the solid/solution interface. The adsorption of Pt(IV) onto Tu–N–SCG–C–A is more likely attributed to physical–chemical process because the standard enthalpy change is less than 80 kJ mol^−1^^67^.

**Table 5:** Thermodynamic parameters of Pt(IV) adsorption on Tu–N–SCG–C–A.

T (K)	$\ln K^\circ$	$\Delta G^\circ$(kJ mol^−1^)	$\Delta S^\circ$(Jmol^−1^ K^-1^)	$\Delta H^\circ$(kJ mol^−1^)
298	3.24	− 8.03	94.39	20.27
308	3.38	− 8.38		
318	3.59	− 8.89		
328	4.01	− 9.93

### Selectivity, desorption and reusability

**Figure 7:**
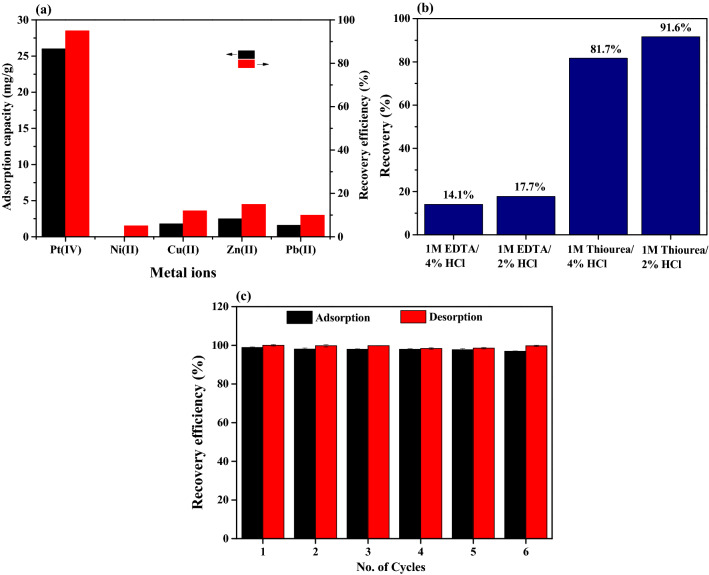
(**a**) Selective adsorption of Pt(IV), (**b**) desorption studies of various solvents and (**c**) regeneration and reusability of Tu–N–SCG–C–A.

In order to evaluate the practical applications, the selective adsorption of noble metals by using Tu–N–SCG–C–A was carried out. As shown in Fig. 7a, the adsorption capacity and recovery efficiency of Pt(IV) observed for the Tu–N–SCG–C–A are the highest among all the metals (i.e., Ni(II), Cu(II), Zn(II) and Pb(II)). With high concentration of chloride, Pt(IV) ions should mostly exist in the forms of [PtCl_6_]^2−^ and [PtCl_5_(H_2_O)]^−^ at the pH = 2, which results in the higher affinity toward the protonated functional groups on the Tu–N–SCG–C–A as compared to other metal ions (positively charged). The desorption efficiency of adsorbed Pt(IV) ions was studied by using various concentrations of EDTA/HCl and thiourea/HCl mixture solution. As shown in Fig. 7b, it can be found that the thiourea can effectively desorb Pt(IV) ions from the Tu–N–SCG–C–A compared to EDTA solution. Moreover, the recovery efficiency is decreased in more acidic solution. This result may be elucidated by weakening the electrostatic interactions between the Pt(IV) ions and the Tu–N–SCG–C–A adsorbents^68^. Among these desorption agents, 1 M thiourea/2% HCl mixture is the superior eluent for Pt(IV) recovery. To reveal the reusability of Tu–N–SCG–C–A, six adsorption–desorption tests were carried out via batch experiments by using 1 M thiourea/2% HCl mixture as an eluent. After each batch, the samples were collected by a strong magnet. As shown in Fig. 7c, the recovery efficiency of Tu–N–SCG–C–A is almost intact after the six successive sorption–desorption cycles. The FTIR spectra (see Fig. S1a) show that the thiourea functional groups observed for used Tu–N–SCG–C–A at 3682, 1580 and 1445 cm^−1^ which are assigned to the stretching vibrations bands of N–H, C=N and N–C–N, respectively, are similar to those of fresh Tu–N–SCG–C–A. However, the lower intensity of peak at 497 cm^−1^ (i.e., Fe–O–Fe) for used Tu–N–SCG–C–A indicates the slight loss of magnetic particles because of the erosion in acidic solution. As observed in TEM and EDS elemental analysis (see Fig. S1b and c), the Fe_3_O_4_ nanoparticles are still existed in the Tu–N–SCG–C–A after six cycles. Although the magnetic response of used Tu–N–SCG–C–A is decreased after six adsorption–desorption runs, the Tu–N–SCG–C–A samples can be separated and recovered by the magnet within 15 s (see Fig. S1d).

### Proposed adsorption mechanism

**Figure 8:**
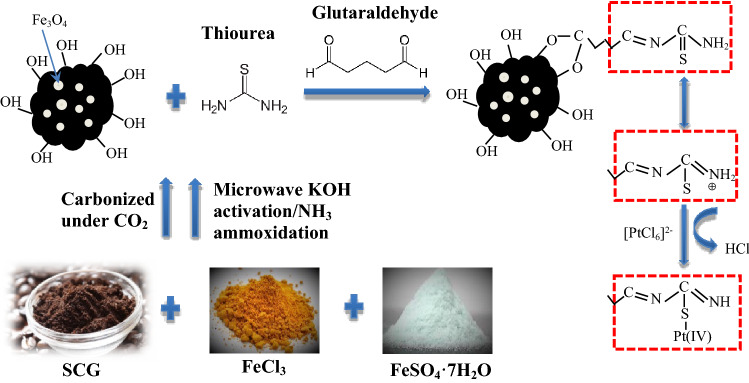
Proposed adsorption mechanism of Pt(IV) onto Tu–N–SCG–C–A.

**Figure 9:**
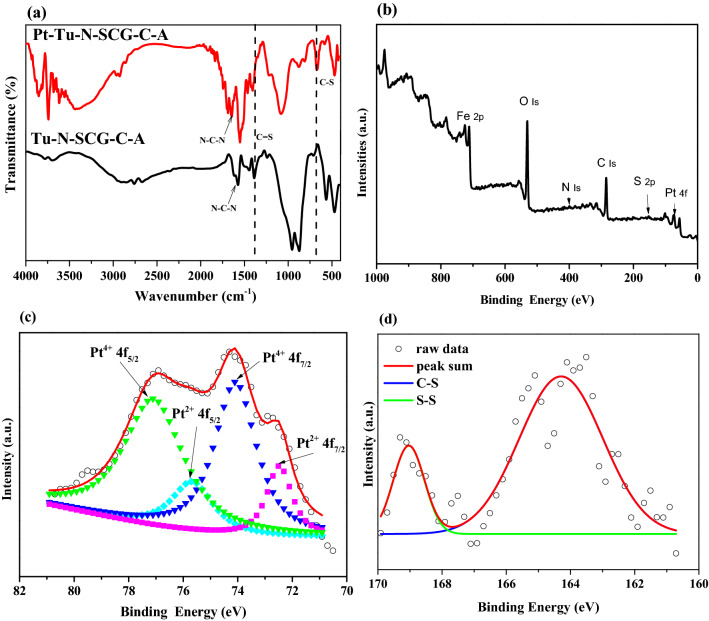
(**a**) FTIR spectra of Tu–N–SCG–C–A and Pt–Tu–N–SCG–C–A, (**b**) XPS survey, (**c**) Pt 4f. and (**d**) S 2*p* spectra of Pt–Tu–N–SCG–C–A.

To further realize the adsorption mechanism of Pt(IV) ions onto Tu–N–SCG–C–A, the spectra of biocarbons after Pt(IV) adsorption (i.e., Pt–Tu–N–SCG–C–A) were measured. As proposed in Fig. 8, glutaraldehyde is used to cross-link amine group (–NH_2_) of thiourea and hydroxyl group (–OH) of Tu–N–SCG–C–A. This result has been discussed in the aforementioned FTIR and XPS analysis. In the previous report^56^, the thioureas have several resonance structures which provide the adsorption sites for Pt(IV) ions. To validate the adsorption mechanisms, FTIR and XPS spectra were carried out to study the interaction between Pt(IV) and thiourea groups. As shown in Fig. 9a, the appearance of typical C–S stretching vibration at 671 cm^−1^ and the decreased intensity of typical C=S stretching absorption at 1378 cm^−1^ suggest the conversion of C=S to C–S in the resonance structure of thiourea. The stretching absorption of N–C–N at 1560 cm^−1^ is shifted to 1648 cm^−1^, indicating that the N–C–N binding may have higher vibration energy after Pt(IV) adsorption. This finding may be due to the transformation of thioureas to the resonance structures. In addition, XPS measurements were used to reveal the adsorption mechanism. In the full survey spectrum of XPS (see Fig. 9b), the Pt 4f. peak can be observed for the Pt–Tu–N–SCG–C–A, indicating the successful accumulation of Pt onto the adsorbents. Further high-resolution Pt 4f. XPS spectrum of Pt–Tu–N–SCG–C–A is shown in Fig. 9c. It can be seen that the Pt 4f_7/2_ spectrum could be deconvoluted into two peaks at 72.5 and 74.3 eV which are ascribed to Pt(IV) and Pt(II) ions, respectively^35,69^. As observed, some of Pt(IV) ions are reduced to Pt(II) after adsorption, which could be attributed to the reduction via C=N and –OH groups on the Pt–Tu–N–SCG–C–A^64^. The S 2p XPS spectrum of Pt–Tu–N–SCG–C–A is deconvoluted and shown in Fig. 9d. Compared to the fitted peaks observed for Tu–N–SCG–C–A (see Fig. 1d), the S 2*p*_3/2_ and S 2*p*_1/2_ peaks (at ca. 164.2 and 169.0 eV, respectively) are shifted to higher binding energies, which may be attributed to the coordination of S and Pt atoms^35^. The aforementioned results confirm the formation pf Pt–S bonding after adsorption. However, the adsorption capacity of Pt(IV) observed for Tu–N–SCG–C–A is ca. 0.22 mmol g^−1^, while the sulfur amount of Tu–N–SCG–C–A is only 0.15 mmol g^−1^, implying that other adsorption mechanism (instead of Pt–S formation) may occur between Tu–N–SCG–C–A and Pt(IV). In view of anionic (i.e., [PtCl_6_]^2−^) and cationic (i.e., Tu–N–SCG–C–A) properties in acidic condition, the electrostatic interaction may also be involved in the adsorption process.

## Conclusions

In summary, the magnetic biocarbons were prepared by carbonizing SCGs in the atmosphere of CO_2_, activating with microwave-assisted KOH process, N-doping treatments by microwave ammoxidation and modifying thiourea by cross-linking via glutaraldehyde in this work. The adsorption kinetics and isotherms observed for adsorption of Pt(IV) were well described by non-linear pseudo second-order model and Freundlich isotherm, respectively, indicating the occurance of multilayer chemical adsorption on the surface of Tu–N–SCG–C–A. The obtained thermodynamic data reveal that the adsorption of Pt(IV) onto the Tu–N–SCG–C–A can happen spontaneously and endothermically. The Tu–N–SCG–C–A adsorbent is able to selectively uptake Pt(IV) ions from simulated wastewater with mixed metal ions. In addition, Pt(IV) ions can be adsorbed and desorbed effectively up to six cyclic runs. From the results of FTIR and XPS spectroscopies, the adsorption mechanisms are attributed to the formation of Pt–S bindings between Pt(IV) and thiourea via the resonance structures. Also, the electrostatic attraction between anionic form of [PtCl_6_]^2−^ and cationic form of Tu–N–SCG–C–A may be involved in the adsorption process. Further grafting more thiourea onto the biocarbons may enhance the adsorption performance. The prepared Tu–N–SCG–C–A adsorbents via energy and time-efficient route are prospective for recovery of noble metals owing to their superior adsorption performance, low-cost and environmental friendliness.

## Supplementary Information

Supplementary Information.

## References

[1] Matsumoto K (2019). Selective and preferential separation of rhodium (III) from palladium (II) and platinum (IV) using a m-phenylene diamine-containing precipitant. Sci. Rep..

[2] Xun DY, Hao H, Sun X, Liu ZW, Zhao FQ (2020). End-of-life recycling rates of platinum group metals in the automotive industry: Insight into regional disparities. J. Clean. Prod..

[3] Ding YJ, Zhang XY, Wu BY, Liu B, Zhang SG (2021). Highly porous ceramics production using slags from smelting of spent automotive catalysts. Resour. Conserv. Recycl..

[4] Grad O (2021). Precious metals recovery from aqueous solutions using a new adsorbent material. Sci. Rep..

[5] Chen Y (2021). Selective recovery of precious metals through photocatalysis. Nat. Sustain..

[6] Vasile E (2021). Making precious metals cheap: A sonoelectrochemical-Hydrodynamic cavitation method to recycle platinum group metals from spent automotive catalysts. Ultrason. Sonochem..

[7] Dong HG (2015). Recovery of platinum group metals from spent catalysts: A review. Int. J. Miner. Process..

[8] Wang J (2021). Pyrometallurgical recovery of zinc and valuable metals from electric arc furnace dust—A review. J. Clean. Prod..

[9] Liu C, Sun SC, Zhu XP, Tu GF (2021). Metals smelting-collection method for recycling of platinum group metals from waste catalysts: A mini review. Waste Manag. Res..

[10] Trinh HB, Lee JC, Srivastava RR, Kim S (2019). Total recycling of all the components from spent auto-catalyst by NaOH roasting-assisted hydrometallurgical route. J. Hazard. Mater..

[11] Liu GQ, Wu YF, Tang AJ, Pan DA, Li B (2020). Recovery of scattered and precious metals from copper anode slime by hydrometallurgy: A review. Hydrometallurgy.

[12] Nguyen VT (2021). Solvometallurgical recovery of platinum group metals from spent automotive catalysts. ACS Sustain. Chem. Eng..

[13] Wongsawa T, Traiwongsa N, Pancharoen U, Nootong K (2020). A review of the recovery of precious metals using ionic liquid extractants in hydrometallurgical processes. Hydrometallurgy.

[14] Yao Y (2020). Alchemy-inspired green paper for spontaneous recovery of noble metals. Small.

[15] Adams MD, Liddell KS, Smith LA (2020). Cyanide-free recovery of metals from polymetallic and refractory gold concentrates by the KellGold process. Hydrometallurgy.

[16] Birich A, Stopic S, Friedrich B (2019). Kinetic investigation and dissolution behavior of cyanide alternative gold leaching reagents. Sci. Rep..

[17] Senthil K, Akiba U, Fujiwara K, Hamada F, Kondo Y (2017). High selectivity and extractability of palladium from chloride leach liquors of an automotive catalyst residue by azothiacalix[4]arene derivative. Hydrometallurgy.

[18] Wang XM, Xu JB, Li L, Li HG, Yang CF (2017). Thiourea grafted PVDF affinity membrane with narrow pore size distribution for Au(III) adsorption: preparation, characterization, performance investigation and modelling. Chem. Eng. J..

[19] Fajar ATN, Kubota F, Firmansyah ML, Gote M (2019). Separation of Palladium(II) and Rhodium(III) using a polymer inclusion membrane containing a phosphonium-based ionic liquid carrier. Ind. Eng. Chem. Res..

[20] Mokhodoeva O, Shkinev V, Maksimova V, Dzhenloda R, Spivakov B (2020). Recovery of platinum group metals using magnetic nanoparticles modified with ionic liquids. Sep. Purif. Technol..

[21] Fajar ATN, Hanada T, Firmansyah ML, Kubota F, Goto M (2020). Selective separation of platinum group metals via sequential transport through polymer inclusion membranes containing an ionic liquid carrier. ACS Sustain. Chem. Eng..

[22] Lanaridi O (2021). Toward the recovery of platinum group metals from a spent automotive catalyst with supported ionic liquid phases. ACS Sustain. Chem. Eng..

[23] Lin M (2021). Mechanism of gold cyanidation in bioleaching of precious metals from waste printed circuit boards. ACS Sustain. Chem. Eng..

[24] Liu JY, Deng Z, Yu HJ, Wang L (2021). Ferrocene-based metal-organic framework for highly efficient recovery of gold from WEEE. Chem. Eng. J..

[25] Yamada M, Kimura S, Gandhi MR, Shibayama A (2021). Environmentally friendly Pd(II) recovery from spent automotive catalysts using resins impregnated with a pincer-type extractant. Sci. Rep..

[26] Wang Z, Kang SB, Won SW (2021). Selective adsorption of palladium(II) from aqueous solution using epichlorohydrin crosslinked polyethylenimine-chitin adsorbent: Batch and column studies. J. Environ. Chem. Eng..

[27] Biswas FB (2021). Highly selective and straightforward recovery of gold and platinum from acidic waste effluents using cellulose-based bio-adsorbent. J. Hazard. Mater..

[28] Tang Y-H, Liu S-H, Tsang DCW (2020). Microwave-assisted production of CO_2_-activated biochar from sugarcane bagasse for electrochemical desalination. J. Hazard. Mater..

[29] Wan ZH (2020). Sustainable impact of tartaric acid as electron shuttle on hierarchical iron-incorporated biochar. Chem. Eng. J..

[30] Rahman MA, Rahman MM, Bahar MM, Sanderson P, Lamb D (2021). Antimonate sequestration from aqueous solution using zirconium, iron and zirconium-iron modified biochars. Sci. Rep..

[31] Li JN (2021). Coffee ground derived biochar embedded O-v-NiCoO_2_ nanoparticles for efficiently catalyzing a boron-hydrogen bond break. Sci. Total Environ..

[32] Norouzi O, Pourhosseini SEM, Naderi HR, Di Maria F, Dutta A (2021). Integrated hybrid architecture of metal and biochar for high performance asymmetric supercapacitors. Sci. Rep..

[33] Osman AI, Farrell C, Al-Muhtase AH, Harrison J, Rooney DW (2020). The production and application of carbon nanomaterials from high alkali silicate herbaceous biomass. Sci. Rep..

[34] Osman AI (2020). Upcycling brewer’s spent grain waste into activated carbon and carbon nanotubes for energy and other applications via two-stage activation. J. Chem. Technol. Biotechnol..

[35] Wang JJ, Li J, Wei J (2015). Adsorption characteristics of noble metal ions onto modified straw bearing amine and thiol groups. J. Mater. Chem. A.

[36] Ma TT (2020). Efficient gold recovery from E-Waste via a chelate-containing porous aromatic framework. ACS Appl. Mater. Interfaces.

[37] Chen SL (2020). Interfacial properties of mercaptopropyl-functionalised silica gel and its adsorption performance in the recovery of gold(I) thiosulfate complex. Chem. Eng. J..

[38] Ronka S, Targonska S (2020). Gold(III) ions sorption on sulfur-containing polymeric sorbent based on 2,2′-thiobisethanol dimethacrylate. Sep. Sci. Technol..

[39] Li LQ (2020). Pit-Induced electrochemical layer dissolution and wave propagation on an Au(111) surface in an acidic thiourea solution. J. Phys. Chem. C.

[40] Chen XM, Xiang Y, Xu L, Liu GJ (2020). Recovery and reduction of Au(III) from mixed metal solution by thiourea-resorcinol-formaldehyde microspheres. J. Hazard. Mater..

[41] Obruca S, Benesova P, Kucera D, Petrik S, Marova I (2015). Biotechnological conversion of spent coffee grounds into polyhydroxyalkanoates and carotenoids. New Biotechnol..

[42] Osman AI (2021). Conversion of biomass to biofuels and life cycle assessment: A review. Environ. Chem. Lett..

[43] Kuo H-C, Liu S-H, Lin Y-G, Chiang C-L, Tsang DCW (2020). Synthesis of FeCo-N@N-doped carbon oxygen reduction catalysts via microwave-assisted ammoxidation. Catal. Sci. Technol..

[44] Kuo H-C, Lin Y-G, Chiang C-L, Liu S-H (2021). FeN@N-doped graphitic biochars derived from hydrothermal-microwave pyrolysis of cellulose for fuel cell catalysts. J. Anal. Appl. Pyrolysis.

[45] Liu S-H, Kuo H-C (2021). Core-shell FeCo N-doped biocarbons as stable electrocatalysts for oxygen reduction reaction in fuel cells. Int. J. Energy Res..

[46] Stylianou M (2018). Converting environmental risks to benefits by using spent coffee grounds (SCG) as a valuable resource. Environ. Sci. Pollut. Res..

[47] Rahmatika AM (2020). Cellulose nanofiber and magnetic nanoparticles as building blocks constructing biomass-based porous structured particles and their protein adsorption performance. ACS Sustain. Chem. Eng..

[48] Liu S-H, Lu J-S, Yang S-W (2018). Highly visible-light-responsive Cu_2_O/rGO decorated with Fe_3_O_4_@SiO_2_ nanoparticles as a magnetically recyclable photocatalyst. Nanotechnology.

[49] Lee JW (2021). Agglomeration-Free Fe_3_O_4_ anchored via nitrogen mediation of carbon nanotubes for high-performance arsenic adsorption. J. Environ. Chem. Eng..

[50] Liu S-H, Chen S-C (2016). Well-dispersed FeN_4_ decorated mesoporous carbons for efficient oxygen reduction in acid media. Carbon.

[51] Liu S-H, Lin W-X (2019). A simple method to prepare g-C_3_N_4_-TiO_2_/waste zeolites as visible-light-responsive photocatalytic coatings for degradation of indoor formaldehyde. J. Hazard. Mater..

[52] Liu S-H, Tang W-T, Yang Y-H (2018). Adsorption of nicotine in aqueous solution by a defective graphene oxide. Sci. Total Environ..

[53] Huang YY (2021). Magnetic phosphorylated chitosan composite as a novel adsorbent for highly effective and selective capture of lead from aqueous solution. J. Hazard. Mater..

[54] Wang L (2018). Microwave-assisted preparation of nitrogen-doped biochars by ammonium acetate activation for adsorption of acid red 18. Appl. Surf. Sci..

[55] Liu SJ (2016). Synthesis of a water-soluble thiourea-formaldehyde (WTF) resin and its application to immobilize the heavy metal in MSWI fly ash. J. Environ. Manag..

[56] Li YC, Tian HY, Xiao CS, Ding JX, Chen XS (2014). Efficient recovery of precious metal based on Au-S bond and electrostatic interaction. Green Chem..

[57] Garole DJ, Choudhary BC, Paul D, Borse AU (2018). Sorption and recovery of platinum from simulated spent catalyst solution and refinery wastewater using chemically modified biomass as a novel sorbent. Environ. Sci. Pollut. Res..

[58] Reffas A (2010). Carbons prepared from coffee grounds by H_3_PO_4_ activation: Characterization and adsorption of methylene blue and Nylosan Red N-2RBL. J. Hazard. Mater..

[59] Muleja AA (2018). Adsorption of platinum ion from aged aqueous solution: application and comparative study between purified MWCNTs and triphenylphosphine MWCNTs. Environ. Sci. Pollut. Res..

[60] Shelimov BN, Lambert JF, Che M, Didillon B (2020). Molecular-level studies of transition metal–support interactions during the first steps of catalysts preparation: Platinum speciation in the hexachloroplatinate/alumina system. J. Mol. Catal. A Chem..

[61] Bediako JK (2020). Recovery of gold via adsorption-incineration techniques using banana peel and its derivatives: Selectivity and mechanisms. Waste Manag..

[62] Saman N, Kamal NAA, Lye JWP, Mat H (2020). Synthesis and characterization of CTAB-silica nanocapsules and its adsorption behavior towards Pd(II) ions in aqueous solution. Adv. Powder Technol..

[63] Chen L (2016). Performance and mechanism of hierarchically porous Ce-Zr oxide nanospheres encapsulated calcium alginate beads for fluoride removal from water. RSC Adv..

[64] Tang JL (2021). Pre-modification strategy to prepare a novel Zr-based MOF for selective adsorption of Palladium(II) from solution. Chem. Eng. J..

[65] He JY (2017). Rapid adsorption of Pb, Cu and Cd from aqueous solutions by beta-cyclodextrin polymers. Appl. Surf. Sci..

[66] Ren G (2017). Chromium (VI) adsorption from wastewater using porous magnetite nanoparticles prepared from titanium residue by a novel solid-phase reduction method. Sci. Total Environ..

[67] Mihailescu M (2019). Gold (III) adsorption from dilute waste solutions onto Amberlite XAD7 resin modified with L-glutamic acid. Sci. Rep..

[68] Chassary P, Vincent T, Marcano JS, Macaskie LE, Guibal E (2005). Palladium and platinum recovery from bicomponent mixtures using chitosan derivatives. Hydrometallurgy.

[69] Azarova YA, Pestov AV, Ustinov AY, Bratskaya SY (2015). Application of chitosan and its N-heterocyclic derivatives for preconcentration of noble metal ions and their determination using atomic absorption spectrometry. Carbohydr. Polym..

