# Supplementary material for: Selective recovery of platinum from spent autocatalyst solution by thiourea modified magnetic biocarbons
Source: Sci Rep. 2021 Sep 29;11:19281. doi: 10.1038/s41598-021-98118-1 (PMC8481563; doi:10.1038/s41598-021-98118-1)
Supplement: Supplementary file 1 — Supplementary Information. [file 41598_2021_98118_MOESM1_ESM.docx]

**Selective recovery of platinum from spent autocatalyst solution by thiourea modified magnetic biocarbons**

Shao-Yi Lo^a^, Wahid Dianbudiyanto^b^, Shou-Heng Liu^a,b,*^

^a^*Department of Environmental Engineering, National Cheng Kung University, Tainan 70101, Taiwan*

*^b^Department of Biology, Faculty of Science and Technology, Universitas Airlangga, Surabaya 60115, Indonesia*

^*^Corresponding authors.

*E-mail address*: [shliu@mail.ncku.edu.tw](mailto:shliu@mail.ncku.edu.tw) (S.-H. Liu)

**Table S1.**

Comparison of Tu-N-SCG-C-A with other reported adsorbents for sorption of Pt(IV) from aqueous media.

| Adsorbents | Surface area  (m^2^ g^-1^) | Pore volume  (cm^3^ g^-1^) | Adsorption capacity  (mg/g) | Experimental conditions | | | | | | Ref. | | |
| --- | --- | --- | --- | --- | --- | --- | --- | --- | --- | --- | --- | --- |
|  |  |  |  | Dosage  (g L^-1^) | Temp.  (K) | Time  (h) | pH | Agitation  (rpm) |  | | | |
| Aminated lignin derivatives | - | - | 42.9 | 0.2 | 303 | 24 | - | - | [1] | |  |  |
| *Providencia vermicola* | - | - | 30.3 | 1.5 | 303 | 3 | 4 | 170 | [2] | |  |  |
| Mesoporous carbon | - | - | 78.0 | 1.0 | 295 | 18 | 1 M HCl | - | [3] | |  |  |
| PEI modified algal biomass | - | - | 115.0 | 0.2 | - | 72 | 2.5 | - | [4] | |  |  |
| Nut shells | 141.18 | - | 38.3 | 4.0 | 298 | 1 | 1.5 | 100 | [5] | |  |  |
| Lysine modified crosslinked chitosan resin | 19.8 | - | 129.3 | 0.1 | 303 | 4 | 1 | 100 | [6] | |  |  |
| Ch-DB18C6 | 14.44 | 0.0153 | 17.6 | 4.0 | 298 | 2 | 2.5 | - | [7] | |  |  |
| Tu-N-SCG-C-A | 13.80 | 0.05 | 41.4-42.8 | 0.25 | 298-328 | 6 | 2 | 175 | This study | | |  |

**Reference**

1. Parajuli, D., Kawakita, H., Inoue, K. & Funaoka, M. Recovery of Gold(III), Palladium(II), and Platinum(IV) by aminated lignin derivatives. *Ind. Eng. Chem. Res.* **45**, 6405-6412 (2006).
2. Xu, H. et al. Competitive biosorption behavior of Pt(IV) and Pd(II) by Providencia vermicola. *RSC Adv.* **7** 32229-32235 (2017).
3. Zalupski, P. R., McDowell, R. & Dutech, G. The adsorption of gold, palladium, and platinum from acidic chloride solutions on mesoporous carbons. *Solvent Extr. Ion Exch.* **32**, 737-748 (2014).
4. Wang, S., Vincent, T., Roux, J. C., Faur, C. & Guibal, E. Pd(II) and Pt(IV) sorption using alginate and algal-based beads. *Chem. Eng. J.* **313**, 567-579 (2017).
5. Morcali, M. H., Zeytuncu, B. & Yucel, O. Platinum uptake from chloride solutions using biosorbents. *Mater. Res.* **16**, 528-538 (2013).
6. Fujiwara, K., Ramesh, A., Maki, T., Hasegawa, H. & Ueda, K. Adsorption of platinum (IV), palladium (II) and gold (III) from aqueous solutions onto l-lysine modified crosslinked chitosan resin. *J. Hazard. Mater.* **146**, 39-50 (2007).
7. Grad, O. et al. Precious metals recovery from aqueous solutions using a new adsorbent material. *Sci. Rep.* **11**, 2016 (2021).

**(b)**


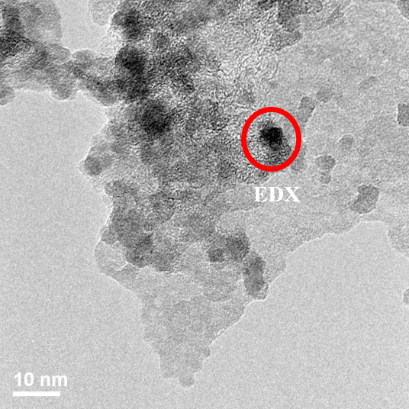


**(d)**

**(c)**


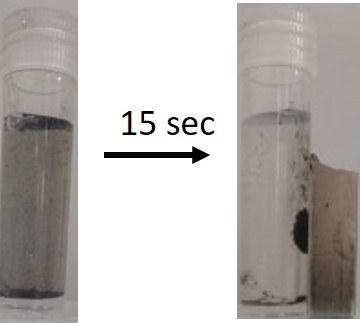

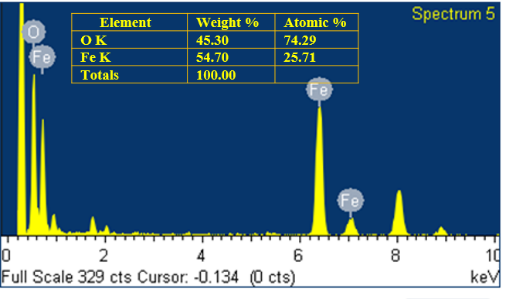


**Figure S1.** (a) FTIR spectra of fresh and used Tu-N-SCG-C-A, (b) TEM image, (c) EDS and (d) photograph of used Tu-N-SCG-C-A which is separated from water by using a permanent magnet.
